# Electro-acupuncture modulated miR-214 expression to prevent chondrocyte apoptosis and reduce pain by targeting BAX and TRPV4 in osteoarthritis rats

**DOI:** 10.1590/1414-431X2024e13238

**Published:** 2024-05-20

**Authors:** Jia He, Jia Zuo, Xiaochen Fan, Zhe Li

**Affiliations:** 1 Department of Traditional Chinese Medical Orthopedics, Xi'an Honghui Hospital, Xi'an Jiaotong University, Xi'an, Shaanxi, China; 2 Department of Acupuncture, Shaanxi Provincial Hospital of Chinese Medicine, Xi'an, Shaanxi, China

**Keywords:** Osteoarthritis, Electro-acupuncture, miR-214, BAX, TRPV4

## Abstract

Osteoarthritis (OA) is a highly prevalent joint disorder characterized by progressive degeneration of articular cartilage, subchondral bone remodeling, osteophyte formation, synovial inflammation, and meniscal damage. Although the etiology of OA is multifactorial, pro-inflammatory processes appear to play a key role in disease pathogenesis. Previous studies indicate that electroacupuncture (EA) exerts chondroprotective, anti-inflammatory, and analgesic effects in preclinical models of OA, but the mechanisms underlying these potential therapeutic benefits remain incompletely defined. This study aimed to investigate the effects of EA on OA development in a rat model, as well as to explore associated molecular mechanisms modulated by EA treatment. Forty rats were divided into OA, EA, antagomiR-214, and control groups. Following intra-articular injection of monosodium iodoacetate to induce OA, EA and antagomiR-214 groups received daily EA stimulation at acupoints around the knee joint for 21 days. Functional pain behaviors and chondrocyte apoptosis were assessed as outcome measures. The expression of microRNA-214 (miR-214) and its downstream targets involved in apoptosis and nociception, BAX and TRPV4, were examined. Results demonstrated that EA treatment upregulated miR-214 expression in OA knee cartilage. By suppressing pro-apoptotic BAX and pro-nociceptive TRPV4, this EA-induced miR-214 upregulation ameliorated articular pain and prevented chondrocyte apoptosis. These findings suggested that miR-214 plays a key role mediating EA's therapeutic effects in OA pathophysiology, and represents a promising OA treatment target for modulation by acupuncture.

## Introduction

Osteoarthritis (OA) is a common joint disorder characterized by degeneration of articular cartilage, bone remodeling, osteophyte formation, synovium inflammation, and meniscal damage (1-3). These changes often lead to chronic pain, stiffness, and disability, especially in the knee joint, which is the most commonly affected joint. While the exact cause of OA remains unclear, inflammation appears to play a key role (4). Treatment options like analgesics and surgery have limitations, so there is increasing interest in non-pharmacological approaches including acupuncture (5-7). Acupuncture involves inserting thin needles into specific body points to stimulate natural healing and well-being (8). Electro-acupuncture (EA) adds small electrical currents to acupoints to enhance effects (9). Although clinical and experimental studies suggest that EA relieves knee pain (5,10,11), its efficacy for OA requires more research.

MicroRNAs (miRNAs) are small noncoding RNA molecules that play important roles in the regulation of various biological processes by regulating the expression of target genes through post-transcriptional mechanisms (12). They have been found to be involved in the progression of OA, and have been shown to play key roles in the degradation of articular cartilage and inflammation in OA (13,14). miR-214 inhibits OA inflammation by downregulating the inhibitor of nuclear factor kappa B kinase subunit beta (IKK-β), disrupting nuclear factor kappa B (NF-κB) signaling (15). Evidence also indicates that EA at PC6 acupoints may regulate miR-214 and protect the heart (16). Thus, EA may treat OA by modulating miR-214.

The present study was designed to investigate the effect of EA treatment on the development of OA in rats, and to explore the potential molecular mechanisms underlying this effect.

## Material and Methods

### Experimental animals

All animal experiments were approved by the Animal Ethics Committee of Xi'an Jiaotong University (approval #2022-1207).

A total of 40 eight-week-old male Sprague Dawley rats (weighing 150-200 g) were obtained from Nanjing Qinglongshan Animal Farm (China) and randomly divided into 4 equal groups of 10 rats each: 1) Osteoarthritis (OA) group: rats received intra-articular injections of 1 mg monoiodoacetate (Sigma Aldrich i2512, USA) in 50 μL saline into the left knee to induce OA; 2) Electroacupuncture (EA) group: rats received monoiodoacetate injections for OA induction and underwent EA treatment; 3) antagomiR-214 group: rats received monoiodoacetate injections, antagomiR-214 (250 μM, 10 μL) intra-articular injections on days 7 and 14 post-OA induction. The antagomiR-214 was designed and synthesized by RiboBio (China); and 4) Control group: rats received intra-articular injections of 50 μL saline.

Rats were housed with a 12-h light/dark cycle and had *ad libitum* access to food and water. After determining the baseline nociceptive responses, behavioral testing was conducted every other day for 21 days after OA induction. At study completion on day 21, rats were euthanized by inhalation anesthesia followed by decapitation.

### EA treatment

This treatment was administered once every day for 21 days, starting on the second day after the induction of OA with the monoiodoacetate injection. The rats in the EA and antagomiR-214 groups received EA treatment at four specific acupoints on the left side of their bodies, near the knee joint: “xiyan” (EX-LE5), “dubi” (ST35), “iangqiu” (ST34), and “xuehai” (SP10). These acupoints are commonly used in acupuncture treatment for OA in humans (17). Before the EA treatment, the rats were anesthetized with 100 mg/kg ketamine (Sigma Aldrich 343099) and 10 mg/kg xylazine (Sigma Aldrich X1126). The rats had their left knee bent at 90 degrees. One stainless steel needle of 0.4 mm diameter (needling depth is 4 mm) was inserted into each of the four acupoints and they were then connected with output terminals of an EA apparatus (ModelG6805-2A, Shanghai Huayi Medical Electronic Apparatus Company, China). The EA needles were inserted to a consistent depth. Alternating strings of dense-sparse frequencies (2/100 Hz EA stimulation, 15 min) were selected as reported previously (18).

### Assessment of pain sensitivity

The weight bearing responses (WBR) measurement is often used in conjunction with the paw withdrawal threshold (PWT) to provide a more comprehensive assessment of an animal's pain sensitivity (19-21). The WBR was determined by comparing the difference in weight bearing ability between the injured (left) and uninjured (right) paws of rats. The amount of weight supported by the hind legs was measured separately for the left and right hind legs, and the difference in weight distribution between the two hind paws was calculated in each rat, using a static weight bearing instrument (IITC Life Science Model-600, USA). These data were then used to calculate the ratio of the left hind paw contribution in total weight bearing.

The PWT measurement was performed as follows: the rats were placed in a transparent cage with metal mesh floor, and von Frey filaments (North Coast Medical Semmes-Weinstein Monofilaments, USA) were applied from underneath the mesh floor to the plantar surface of the operated paw. The von Frey filament stimulated the sole of the injured foot, and its stimulation intensity gradually increased (2-15 g). The rapid reactions of the rat, such as clawing, lifting, and licking its feet, were recorded by a computer. This process was repeated three times with a 20-min interval between each test to obtain an average value.

### TUNEL assay

The terminal deoxynucleotidyl transferase dUTP nick end labeling (TUNEL) assay, which is used to detect DNA fragmentation and cell apoptosis, was performed using a TUNEL detection kit according to the manufacturer's instructions (Abcam ab66110, UK). Briefly, knee joint sections were incubated with 15 µg/mL of proteinase K (15 min, 23°C) and then treated with TdT enzyme (90 min, 37°C), which adds the labeled nucleotide to the ends of the fragmented DNA. After washing in phosphate-buffered saline, the slides were examined by fluorescence microscopy. The fluorescent images of cryosections were recorded using an Olympus DP70 digital camera coupled to an Olympus IX71 inverted microscope (Japan). The fluorescent images were measured using ImageJ software (NIH, USA). The rate of apoptosis in articular cartilage was defined as the ratio of the summed squared pixel intensity of TUNEL-positive staining (green-stained cells) to that of the background regions.

### miRNA microarray analysis

Total RNA was extracted from the articular cartilage tissues of control and OA rats using TRIzol (ThermoFisher 15596026, USA) and PureLink™ miRNA Isolation Kit (ThermoFisher K157001) according to manufacturer's instructions. The quantity and quality of the RNA was then measured using a NanoDrop 1000 spectrophotometer (Youpu, China). The samples were labeled with a fluorescent dye (miRCURY™Hy3™/Hy5™ Power labeling kit) and hybridized to a miRCURY LNA array (Exiqon, Denmark). After washing, the array was scanned using an Axon GenePix 4000 B microarray scanner (Axon Instruments, USA), and the data was analyzed using the GenePix Pro 6.0 program (Axon Instruments). Data were normalized by median normalization. After normalization, the miRNAs that were significantly differentially expressed between the control and OA groups were identified by Volcano Plot filtering (Origin 2021, USA).

### Quantitative real-time polymerase chain reaction (qRT-PCR)

The rats in the OA, EA, and antagomiR-214 groups were euthanized at 7, 14, and 21 days post-OA, total RNA from the articular cartilage tissues was extracted with TRIzol (ThermoFisher 15596026) according to manufacturer's instructions, and cDNA was synthesized from 1 μg of total RNA using the TaKaRa RNA PCR Kit (Takara, Japan). cDNA was used as a template for reverse transcription polymerase chain reaction analysis amplification (Mastercycler Gradient, Germany) to observe the miRNAs expression by using the miRNAs-specific primers for miR-214 (Ribobio, China). The expression levels of the miRNAs were normalized to the expression of the U6 gene. The primers used to amplify U6 were 5'-CTCGCTTCGGCAGCACA-3' (forward) and 5'-AACGCTTCACGAATTTGCGT-3' (reverse). All reactions were performed in triplicate. The relative expression of miRNAs was normalized to U6. Data were analyzed by using the 2^−ΔΔCt^ method.

### Luciferase reporter assay

The miR-214 mimic or inhibitor and corresponding negative control (NC) were designed and synthesized by RiboBio. The sequence of miR-214 mimic was as follows: 5'-UGCCUGUCUACACUUGCUGUGC-3'. The sequence of miR-214 inhibitor was as follows: 5'-ACUGCCUGUCUGUGCCUGCUGU-3. The sequence of NC mimic was as follows: 5'-CUCACCAAAAACCCUAUGGUAG-3'. The sequence of NC inhibitor was as follows: 5'-UCUACUCUUUCUAGGAGGUUGUGA-3'. Dual-luciferase reporter assay was performed according to the manufacturer's instructions. The fragment of the 3'-untranslated region (3'-UTR) of BAX and TRPV4 (wild-type or mutant) was amplified and cloned into the pMIR-REPORT luciferase vector (ThermoFisher AM5795). For the luciferase assay, chondrocyte-like ATDC5 cells (ATCC, USA) were grown to approximately 60% confluence in 24-well plates and co-transfected with 100 nM miR-214 mimic/inhibitor and 0.8 μg pMIR-REPORT luciferase reporters (containing wild-type or mutant 3'-UTR of BAX and TRPV4). After 48 h of transfection, luciferase activity was assessed according to the dual-luciferase reporter assay protocol (Promega, USA). Each experiment was repeated three times.

### Western blot analysis

The required total proteins were isolated from the articular cartilage tissues of the rats in the OA, EA, antagomiR-214, and control groups, which were lysed with RIPA reagents (ThermoFisher 89901) and complete protease inhibitors (11697498001, Roche, Switzerland) on ice. The protein level of the supernatant was determined using the bicinchoninic acid assay (Abcam ab102536). For western blot analysis, the lysate proteins were resolved by sodium dodecyl sulfate polyacrylamide gel electrophoresis and blotted onto nitrocellulose membranes to test the binding of the antibodies. The first antibodies were: BAX (1:1000; Abcam ab32503), TRPV4 (1:1000; ThermoFisher PA5-77319), and β-actin (1:5000; Abcam ab8227). The secondary antibody was Goat Anti-Rabbit IgG H&L (1:5000; Abcam ab150077). Experiments were repeated three times.

### Statistical analysis

The data are reported as means±SD. Statistical analysis was performed using Prism software (GraphPad Software, USA). Data normality was assessed using the Shapiro-Wilk test. For comparisons between two independent groups with normally distributed data, the Student's *t*-test was utilized. In cases where the data did not follow a normal distribution, the Mann-Whitney test was employed for comparison. When there were more than two groups, statistical comparisons were performed using one-way analysis of variance (ANOVA) followed by pairwise comparisons with Benjamini-Hochberg corrections. A significance level of P<0.05 was considered statistically significant.

## Results

### EA treatment relieved pain in OA rats

**Figure 1:**
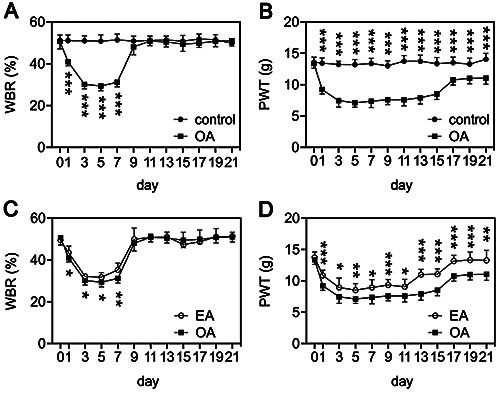
Effects of electroacupuncture (EA) on mechanical allodynia in osteoarthritis (OA) rats. **A** and **C**, Percent contribution of weight bearing (WBR) by the injured (left) hind paw in total weight bearing measured in control, OA, and EA groups. **B** and **D**, Mechanical paw withdrawal threshold (PWT) (in grams) was measured in control, OA, and EA groups. Data are reported as means±SD of n=10 rats. *P<0.05; **P<0.01; ***P<0.001. Student's *t*-test was utilized when the data followed a normal distribution at each time point (day 0 to day 21) and the Mann-Whitney test was utilized to compare non-normally distributed data.

PWT and WBR are commonly used to assess pain sensitivity in animals (19-21). At day 1 post-monoiodoacetate injection, the left hind paw contribution in total weight bearing and the mechanical PWT were significantly decreased in the OA group compared with that in the control group (Figure 1A and B). These reductions persisted for at least 7 and 21 days, respectively. The EA treatment was able to significantly reverse these reductions in weight bearing capacity and PWT (Figure 1C and D), suggesting that it may have pain-relieving effects in OA rats.

### EA treatment protected the articular cartilage of OA rats

**Figure 2:**
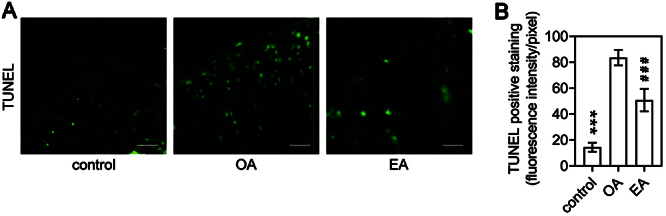
**A**, TUNEL staining of articular cartilage sections from rats in the control, osteoarthritis (OA), and electroacupuncture (EA) groups. Scale bar: 100 μm. **B**, Quantitation of TUNEL-positive staining in outer border cartilages of rat knees. Data are reported as means±SD of n=10 rats. ***P<0.001; ^###^P<0.001 compared to OA (one-way ANOVA).

Chondrocyte apoptosis is increased in OA cartilage (22). The TUNEL assay (Figure 2A and B) showed that the number of TUNEL-positive chondrocytes was increased in the joints of OA rats compared to control rats, indicating an increase in cell apoptosis in the OA cartilage. The EA treatment was able to significantly reduce the number of TUNEL-positive chondrocytes in the OA rats, suggesting that it may have protective effects on the articular cartilage by preventing chondrocyte apoptosis.

### Aberrant expression of miRNAs in injured articular cartilage following EA treatment

**Figure 3:**
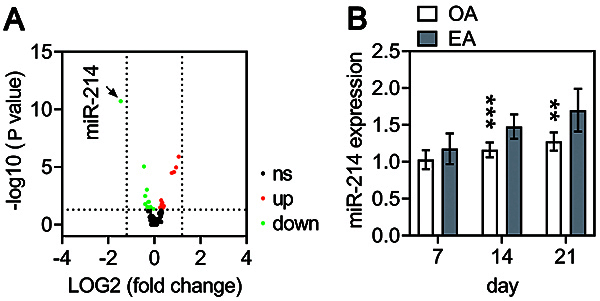
Identification of miRNAs levels in injured articular cartilage of osteoarthritis (OA) rats with or without electroacupuncture (EA) treatment. **A**, Volcano plot representing 160 miRNAs in injured articular cartilage with the log2-fold change plotted against the negative log10 adjusted P value for OA group compared to control groups (at 21 days post-OA induction). Red and green dots indicate upregulated and downregulated miRNA compared to the control group (adjusted P value <0.05). Horizontal (adjusted P value <0.05) and vertical lines (fold change >1.2) show the thresholds used for analysis. ns, non-statistical difference. **B**, miR-214 expression was quantified in injured articular cartilage of rats in OA and EA groups at 7, 14, and 21 days post-OA induction. Data are reported as means±SD of n=10 rats. **P<0.01; ***P<0.001 compared to EA (Student's *t*-test).

The expressions of a large set of miRNAs were altered between control and OA rats. Among these, miR-214 was the most significantly down-regulated (Figure 3A). miR-214 expression was significantly up-regulated in injured articular cartilage of EA group compared with the OA group at 14 and 21 days post-EA (Figure 3B).

### miR-214 suppressed BAX expression by directly targeting its 3'-UTR

**Figure 4:**
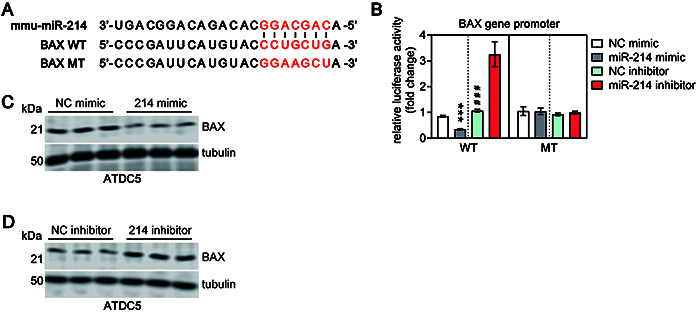
**A**, The 3'-UTR region of BAX mRNA containing the wild type (WT) or mutant (MT) binding site for miR-214. **B**, The relative luciferase activity of BAX WT or MT 3'-UTR in ATDC5 cells following transfection with miR-214 mimic/inhibitor or corresponding negative control (NC). Data are reported as means±SD of three replicates. ***P<0.001 compared to NC mimic; ^###^P<0.001 compared to miR-214 inhibitor (Student's *t*-test). **C** and **D**, Western blot was conducted to detect the protein level of BAX in ATDC5 cells following transfection with miR-214 mimic/inhibitor or corresponding NC. Tubulin was used as an internal control.

The expression of BAX has been implicated in the susceptibility of chondrocytes to apoptosis (23). We found that miR-214 was upregulated in injured articular cartilage of OA rats after EA treatment, and a previous study revealed that halothane increases neuronal cell death vulnerability by downregulating miR-214 and upregulating BAX (24). Bioinformatic analysis showed that BAX gene might be a potential target of miR-214 and the target site located in the 3'-UTR (Figure 4A). Therefore, we constructed both a wild type (WT) and a mutant type (MT) of firefly luciferase reporters containing the 3'-UTR of BAX. The reporters were co-transfected with either miR-214 mimic/inhibitor or NC mimic/inhibitor to chondrocyte-like ATDC5 cells, and the luciferase activity was measured. We observed that miR-214 mimic decreased the relative luciferase activities in the presence of wild-type 3'-UTR, whereas miR-214 mimic did not inhibit the luciferase activity of the reporter vector containing 3'-UTR of BAX with mutations in the miR-214-binding site (Figure 4B). To further confirm that BAX was negatively regulated by miR-214, BAX expression was analyzed by western blot analysis. We found that BAX levels were significantly downregulated after transfection with miR-214 mimic, compared with the NC (Figure 4C). By contrast, transfection with the miR-214 inhibitor enhanced BAX expression (Figure 4D). Taken together, these results indicated that miR-214 inhibited the expression of the apoptosis-related protein BAX by directly targeting its 3'-UTR, which also suggested that the regulation of miR-214 and BAX might be involved in the progression of OA and that EA treatment may have an effect on this regulation.

### miR-214 suppressed TRPV4 expression by directly targeting its 3'-UTR

**Figure 5:**
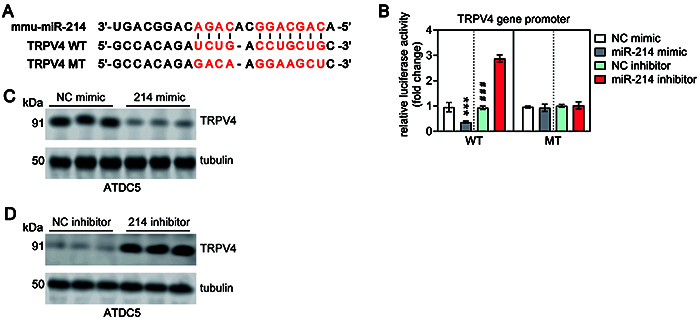
**A**, The 3'-UTR region of TRPV4 mRNA containing the wild type (WT) or mutant type (MT) binding site for miR-214. **B**, Relative luciferase activity of TRPV4 WT or MT 3'-UTR in ATDC5 cells following transfection with the miR-214 mimic/inhibitor or corresponding negative control (NC). Data are reported as means±SD of three replicates. ***P<0.001 compared to NC mimic; ^###^P<0.001 compared to miR-214 inhibitor (Student's *t*-test). **C** and **D**, Western blot was conducted to detect the protein level of TRPV4 in ATDC5 cells following transfection with miR-214 mimic/inhibitor or corresponding NC. Tubulin was used as an internal control.

TRPV4 has been implicated in the development of mechanical hyperalgesia, which is a type of increased sensitivity to pain that occurs in response to mechanical stimuli such as pressure or touch (25). This condition is often observed in individuals with chronic pain conditions, such as osteoarthritis. Our results of bioinformatics analysis also showed that TRPV4 might be a target gene of miR-214 and the target site was located in the 3'-UTR (Figure 5A). To determine whether miR-214 directly repressed the TRPV4 gene transcription, the WT or MT of 3'-UTR region of TRPV4 mRNA was constructed and inserted into the pMIR-REPORT luciferase vector. We found that miR-214 mimic efficiently suppressed the luciferase activity compared to NC mimic, but miR-214 inhibitor significantly enhanced the luciferase activity compared with the NC inhibitor (Figure 5B). To further confirm this result, we performed western blot to detect the TRPV4 protein expression. The results showed that miR-214 mimic significantly inhibited TRPV4 protein expression, but the TRPV4 mRNA protein expression was significantly upregulated by miR-214 inhibitor compared with NC inhibitor (Figure 5C and D). These data indicated that miR-214 inhibited pain-related TRPV4 gene expression by directly targeting its 3'-UTR. It is worth noting that these findings are based on *in vitro* experiments and further research is needed to confirm and fully understand the role of miR-214 and TRPV4 in the development of mechanical hyperalgesia.

### EA attenuated BAX and TRPV4 expression by upregulating miR-214 in injured articular cartilage of OA rats

**Figure 6:**
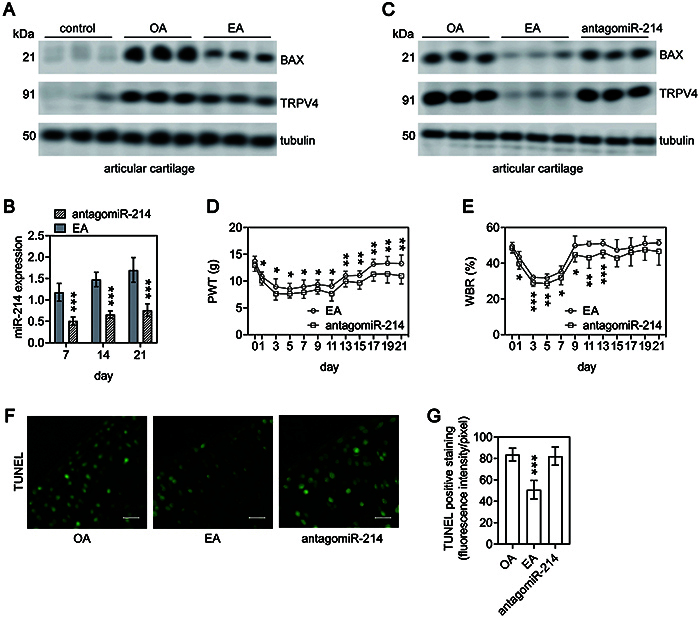
The effects of electroacupuncture (EA) treatment in osteoarthritis (OA) rats were based on miR-214. **A**, Western blot was conducted to detect the protein level of BAX and TRPV4 in the injured articular cartilage of rats of the control, OA, and EA groups. Tubulin was used as an internal control. **B**, miR-214 expression was quantified in injured articular cartilage of rats in EA and antagomiR-214 groups at 7, 14, and 21 days post-OA induction. **C**, Western blot was conducted to detect the protein level of BAX and TRPV4 in the injured articular cartilage of rats of the OA, EA, and antagomiR-214 groups. Tubulin was used as an internal control. **D** and **E**, Percent of the injured (left) hind paw contribution in mechanical paw withdrawal threshold (PWT) (**D**) and total weight bearing (WBR) (**E**). **F**, TUNEL staining in sections of articular cartilage in the proximal tibia from the knee joints of the three groups. Scale bar: 100 μm. **G**, Quantitation of TUNEL-positive staining. Data are reported as means±SD of n=10 rats. **B** and **E**: *P<0.05, **P<0.01, ***P<0.001 compared to EA group; **D**: *P<0.05, **P<0.01 compared to antagomiR-241 group. **G**: ***P<0.001 compared to OA and antagomiR-214 groups (Student's *t*-test).

Based on the above results, our data indicated that EA resulted in miR-214 upregulation in OA rats and miR-214 inhibited BAX and TRPV4 expression by directly targeting its 3'-UTR *in vitro*. To further investigate whether EA suppressed BAX and TRPV4 expression in OA rats by upregulating miR-214, we performed western blot analysis to determine BAX and TRPV4 expressions in injured articular cartilage of OA rats. The results showed that BAX and TRPV4 were upregulated in injured articular cartilage of OA rats compared with control rats and were significantly downregulated following EA treatment (Figure 6A). However, the downregulation of miR-214 by antagomiR-214 (Figure 6B) markedly rescued the suppressive effects of EA on BAX and TRPV4 expressions in OA rats (Figure 6C). Furthermore, the benefits of EA treatment on articular pain (Figure 6D and E) and chondrocyte apoptosis (Figure 6F and G) in OA rats were reversed by antagomiR-214 injection.

## Discussion

In this study, we assessed the effect of EA treatment in a rat model of OA. Our data demonstrated that EA therapy alleviated pain and prevented chondrocyte apoptosis in OA rats. Furthermore, we confirmed that the miR-214 expression was markedly increased following EA treatment, as were the apoptosis protein BAX and pain protein TRPV4, two functional targets of miR-214 *in vitro* and *in vivo*. These data might contribute to a better understanding of the mechanism involved in EA treatment on OA at the miRNA level and provide a new therapeutic strategy for OA.

Acupuncture is commonly used in Traditional Chinese Medicine to treat various conditions including pain. There are various modalities of acupuncture, including manual acupuncture, EA, auricular acupuncture, and moxibustion. Among these, EA provides a standardized, consistent stimulus that can be modulated according to treatment needs and patient response in real-time (8,26,27). EA treatment at specific acupoints has been shown to have a therapeutic effect on OA in animal models (17,28,29). The analgesic effects of EA are believed to be mediated by neurotransmitters and neurohormonal modulation. EA also appears to have anti-inflammatory properties (8,26,27). In recent years, a great number of studies have reported the mechanisms of cell survival by EA. The Bcl-2 family and caspase family commonly participate in mitochondrial apoptotic pathway (30,31). Bcl-2 family proteins are pivotal moderators of apoptosis, and the expressions of Bcl-2 and BAX are closely correlated with cell survival. It has been reported that acupuncture treatment results in a significant downregulation of BAX, but an upregulation of Bcl-2 in rabbit knee OA (32). In this study, we found that EA treatment prevented chondrocyte apoptosis in OA rats, as evidenced by a reduction in DNA fragmentation as detected by TUNEL staining. Our data further confirmed that EA significantly downregulated the expression levels of BAX in injured articular cartilage of OA rats. The present findings suggested that EA could protect articular cartilage degeneration during OA progression, possibly by regulating apoptosis-related proteins. However, the possible molecular mechanisms need further research to be better understood.

The mechanism underlying EA-relieved chronic pain in OA also remains elusive. Nociceptors are sensory neurons that detect potentially harmful stimuli and transmit this information to the central nervous system, leading to the perception of pain (33). Transient receptor potential (TRP) channels (33,34) are a group of ion channels that play a key role in the detection of noxious stimuli and activation of nociceptors. Several TRP channels have been implicated in the perception of pain, including TRPV1, TRPV4, TRP melastatin 8 (TRPM8), and TRP ankyrin 1 (TRPA1). TRPV4, in particular, has been suggested as a potential target for the treatment of mechanical hyperalgesia and other chronic pain conditions (25,35-37). Previous research has shown that TRPV4 plays a role in the mechanotransduction process in chondrocytes (38), the cells that produce and maintain cartilage in joints, in response to injurious mechanical loading. In the present study, we confirmed that TRPV4 expression was increased in injured articular cartilage of OA rats, while EA treatment reversed this increase.

Recent studies have shown that OA causes aberrant expression of many miRNAs and modulates the expression of their target genes (13,14). There is some evidence to suggest that acupuncture may modulate miRNA expression (39) and have therapeutic effects in OA. Inspired by these studies, we performed microarray analysis to identify miRNA levels following EA treatment in the articular cartilage of OA rats. Our findings showed that a large number of miRNAs were expressed differently in the articular cartilage of OA rats after EA treatment, with miR-214 being one of the most significantly upregulated. Therefore, we investigated the miR-214 function by predicting miR-214 putative targets. The luciferase reporter assay showed that miR-214 inhibited BAX and TRPV4 by directly targeting their 3'-UTR. The analysis of qRT-PCR and western blot confirmed that miR-214 controlled the expressions of BAX and TRPV4 in chondrocyte-like ATDC5 cells. Moreover, the downregulation of miR-214 by antagomiR-214 significantly reversed the suppressive effects of EA on BAX and TRPV4 expression in injured articular cartilage, confirming the benefits of EA treatment on pain relief and chondrocyte protection in OA rats. However, when miR-214 was downregulated using an antagomiR, the suppressive effects of EA on BAX and TRPV4 expression were rescued, and the benefits of EA treatment on articular pain and chondrocyte apoptosis in OA rats were reversed.

In conclusion, our results provided new insights into the mechanism of EA treatment that ameliorate articular pain and prevent chondrocyte apoptosis in OA rats. In addition, we found that miR-214 was markedly upregulated in OA rats following EA treatment. Most importantly, we validated BAX and TRPV4 as two functional targets of miR-214 *in vitro* and *vivo*, and showed that EA attenuated OA-induced TRPV4 and BAX upregulation in injured articular cartilage via upregulating miR-214. Our findings might contribute to a better understanding of the EA mechanism on OA treatment and miR-214 could be a potential therapeutic intervention for OA.
